# Effect of Turkish propolis extracts on proteome of prostate cancer cell line

**DOI:** 10.1186/1477-5956-9-74

**Published:** 2011-12-07

**Authors:** Yaşam Barlak, Orhan Değer, Meltem Çolak, Senem Ceren Karataylı, Abdurrahman Mithat Bozdayı, Fulya Yücesan

**Affiliations:** 1 School of Health Sciences, Gümüşhane University, Gümüşhane, 29100, Turkey; 2 Department of Biochemistry, Faculty of Medicine, Karadeniz Technical University, Trabzon, 61080, Turkey; 3 Institute of Hepatology, Faculty of Medicine, Ankara University, Ankara, 06100, Turkey

**Keywords:** Propolis, PC-3 Prostate Cancer Cell Line, Proteome, SELDI-TOF MS

## Abstract

**Background:**

Propolis is a natural, resinous hive product that has several pharmacological activities. Its composition varies depending on the vegetation, climate, season and environmental conditions of the area from where it was collected. Surface enhanced laser desorption ionization time of flight mass spectrometry (SELDI-TOF MS) is a proteomic approach which has been used in cancer proteomics studies. Prostate cancer is one of the most commonly diagnosed cancers in men. It has shown that nutritional supplements rich in polyphenolic compounds such as propolis play a significant role in prostate cancer chemoprevention. The aim of this study is to evaluate if protein expression profile in PC-3 prostate cancer cell lines could be differentiated when incubated with dimethyl sulfoxide and water extracts of Turkish propolis.

**Results:**

The antioxidant potentials of dimethyl sulfoxide and water extracts of propolis were found in correlation with the amount of total phenolic compounds of them. Dimethyl sulfoxide and water extracts of propolis of 20 μg/mL reduced the cell viability to 24.5% and 17.7%, respectively. Statistically significant discriminatory peaks between control PC-3 cells and dimethyl sulfoxide extract of propolis-treated PC-3 cells were found to be the proteomic features at m/z 5143, 8703, 12661, 20184 and 32794, detected by CM10 ProteinChip, and the peak at m/z 3772, detected by Q10 ProteinChip. Between control PC-3 cells and water extract of propolis-treated PC-3 cells, statistically significant discriminatory peaks were found to be the proteomic features at m/z 15846, 16052 and 24658, detected by CM10 ProteinChip and the peaks at m/z 10348, 10899 and 11603, detected by Q10 ProteinChip.

**Conclusions:**

It was concluded that dimethyl sulfoxide and water extracts of Turkish propolis may have anti-proliferative activity through differentiating protein expression profile in PC-3 prostate cancer cell lines along with their antioxidant capacity.

## Background

Propolis is a natural, resinous hive product that honeybees manufacture by mixing their own waxes and salivated secretions with resins collected from the resin from the cracks in the bark of trees and leaf buds [1-4]. The chemical composition of propolis depends on the vegetation, climate, season and environmental conditions of the area from where it was collected [1,5]. It is mainly composed of resin and vegetable balsam (50%), wax (30%), essential and aromatic oils (10%), pollen (5%), and other various substances including organic compounds and minerals (5%) [3,5,6]. Organic compounds that are identified in different propolis samples are fatty and phenolic acids and esters, substituted phenolic esters, flavonoids (flavones, flavanones, flavonols, dihydroflavonols, chalcones), terpenes, β-steroids, aromatic aldehydes and alcohols, sesquiterpenes, naphtalene and stilbene derivatives [7,8]. Propolis has a long history for being used in folk medicine and includes various biological activities such as anti-microbial, anti-tumor, anti-bacterial, anti-fungal, anti-viral, anti-inflammatory, anti-oxidant, anti-cancer, anti-protozoan, cariostatic, hepatoprotective and immunostimulant etc. [1,2,8,9]. Biological actions of propolis are generally attributed to phenolic compounds in its content [5,8]. Anti-tumoral activity of propolis might be attributed to a single substance, or to synergistic effects of several compounds, or to potential metabolites. On the other hand, poor activity might be due to antagonistic effects of its components or the absence or low concentration of active substances [10]. Propolis has been used in food and beverages, and is thought to improve health and prevent diseases such as inflammation, heart disease, diabetes and cancer [2,11,12].

The term ''proteome'' describes ''all proteins expressed by the genome of a cell, a tissue or an organism'' and was introduced by Marc Wilkins [13,14]. In contrast to the genome, the proteome is dynamic collection of proteins that represents both the intrinsic genetic programme of the cell and and the impact of its immediate environment [14,15]. Thus, the proteome provides a more realistic view of a biological status compared with the genome. Proteome is expected to be more useful than genome to evaluate disease presence, progression and response to treatment [14]. Proteomics is a scientific approach used to elucidate all protein species within a cell or tissue [16]. During the transformation of a healthy cell into a neoplastic cell, distinct changes occur at the protein level such as altered expression, differential protein modification, changes in specific activity, and aberrant localization. Researchers propose to identify and understand these changes by cancer proteomics studies [15]. Recently, new strategies that facilitate proteomic analysis, such as, the surface-enhanced laser desorption ionization time-of-flight mass spectrometry (SELDI-TOF-MS) have been introduced [17]. This technology uses protein chips made of a variety of chromatographic surfaces to capture proteins from a complex mixture that are subsequently ionized and detected by TOF MS and provides a peak whose intensity is relatively quantitative and reproducible measure of a particular protein [16,18,19]. Changes in the protein peaks, or m/z ratios within the spectra, can be used to identify protein changes that may underlie in pathophysiological processes. Alternatively, SELDI can also be used to generate peptide mass fingerprint (PMFs) from a complex protein sample, which is then compared to theoretical PMFs of known and DNA sequence-derived whole proteins contained within databases [16]. SELDI-TOF MS allows protein profiling from a variety of complex biological materials such as serum, blood, plasma, intestinal fluid, urine, cell lysates and cellular secretion products [19]. SELDI has several advantages because of its high throughput, versatility, ease of use. It is rapid, reproducible, highly sensitive (detection limit in the femtomolar range) and readily adaptable to a diagnostic format [14,15]. But it is unsuitable for high molecular weight proteins (> 100 kDa); limited to detection of bound proteins; lower resolution and mass accuracy [14].

Prostate cancer is one of the most commonly diagnosed cancers in men and develops in nearly 30% of all men above the age of 50 years [20,21]. Prostate cancer may metastasize to other parts of the human body, especially bones and lymph nodes [21]. The factors that determine the risk of developing clinical prostate cancer have been identified as increasing age, ethnicity, and heredity [22]. Multiple genes and additional environmental factors such as diet and inflammation are also involved in the pathogenesis of prostate cancer. Furthermore, marked geographic variations have been observed in the incidence of clinical prostate cancer [23]. In the treatment of prostate cancer, many chemotherapeutic agents such as Eulexin, Flutamide and Nilandron have been developed. However, undesirable side effects such as urinary incontinence and erectile dysfunction can reduce the therapeutic efficacy of prostate cancer [21]. Recent studies have shown that, nutritional supplements, such as Vitamin E, Vitamin D, soybean, green tea, turmeric, vegetables and fruits or plant extract rich in polyphenolic compounds, play a significant role in prostate cancer chemoprevention [20,24].

The major aim of this study is to evaluate if protein expression profile in PC-3 prostate cancer cell lines could be differentiated when incubated with dimethyl sulfoxide (DMSO) and water extracts of Turkish propolis by SELDI-TOF MS.

## Results

### Total Phenolic Contents and Antioxidant Potentials of Propolis Extracts

Total polyphenol content, total flavonoid content, Ferric reducing antioxidant potential (FRAP) and total antioxidant capacities (TAC) of dimethyl sulfoxide extract of propolis (DEP) and water extract of propolis (WEP) were determined as mg gallic acid (GA)/g propolis, mg quercetin (Q)/g propolis, mg trolox (Tro)/g propolis and mmoltrolox (Tro)/100 g propolis, respectively. Results were shown in Table 1. The antioxidant potentials of DEP and WEP were found to correlate with the amount of total phenolic compounds in them.

**Table 1:** Total Phenolic Contents and Antioxidant Potentials of Propolis Extracts (mean ± standard deviation)

	DEP	WEP
**Total polyphenol content** **(mg GA/g propolis)**	48.7 ± 7.8	9.2 ± 0.5
**Total flavonoid content** **(mg Q/g propolis)**	13.0 ± 1.6	2.1 ± 1.5
**Ferric Reducing Antioxidant Potential (FRAP)** **(mg Tro/g propolis)**	59.5 ± 17.3	24.1 ± 6.1
**Total antioxidant capacities** **(mmolTro/100 g propolis)**	8.8 ± 3.0	5.0 ± 0.8

DEP refers to DMSO (100%) extract of propolis; WEP refers to water extract of propolis.

### PC-3 Cell Viability and Anti-proliferative Effect of Propolis Extracts

The cell viability of DMSO solutions-, DMSO extract of propolis (DEP)- and water extract of propolis (WEP)- treated PC-3 cells were determined. Results shown in Table 2. DMSO and water extracts of propolis of 20 μg/mL, and 0.008% DMSO solution reduced the cell viability to 24.5%, 17.7% and 75.0%, respectively.

**Table 2:** PC-3 Cell Viability and Anti-proliferative Effect of Propolis Extracts.

Propolis Extracts/DMSO Solutions	Cell Viability %
20 μg/ml DEP	24.5 ± 2.8
10 μg/ml DEP	35.1 ± 16.9
5 μg/ml DEP	50.0 ± 20.5
% 0,008 DMSO	75.0 ± 16.6
% 0,004 DMSO	71.8 ± 21.4
% 0,002 DMSO	72.7 ± 19.3
20 μg/ml WEP	17.7 ± 11.9
10 μg/ml WEP	26.4 ± 8.5
5 μg/ml WEP	29.8 ± 12.8

DEP refers to DMSO (100%) extract of propolis; WEP refers to water extract of propolis.

### Expression Difference Mapping between DMSO extract of propolis-treated and untreated PC-3 cancer cell lines by SELDI-TOF-MS

**Figure 1:**
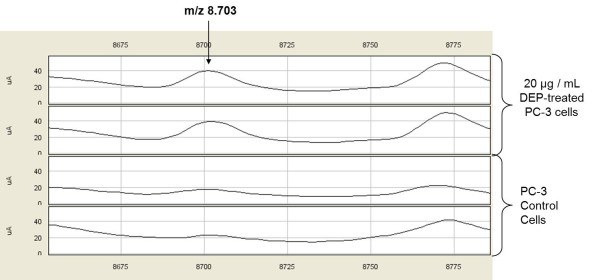
**SELDI-TOF mass spectra of DEP-treated and untreated PC-3 cells by CM10 ProteinChip**. Protein peaks of the peak 8703 m/z, and that positively correlated with DEP-treated PC-3 cells.

**Figure 2:**
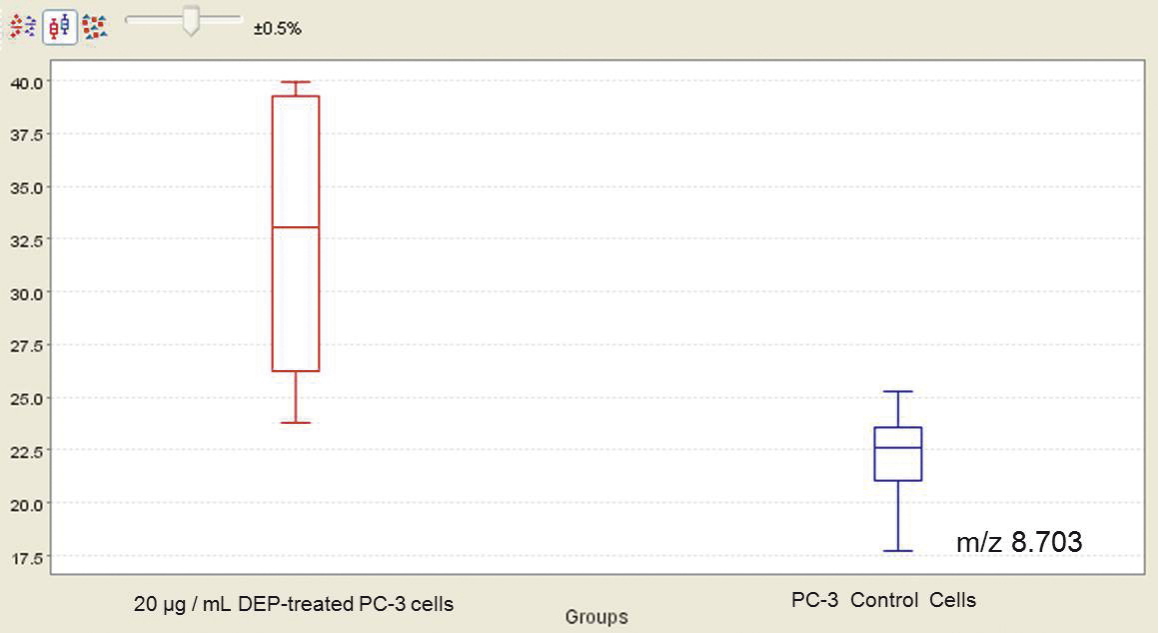
**Box plot displays of intensity levels of 8703 m/z betweenDEP-treated and untreated PC-3 cells by CM10 ProteinChip**. The comparison of DEP-treated PC-3 cell lysates and control PC-3 cell lysates are performed by using non-parametric Mann Whitney method with Ciphergen Express software, version 3.0.

As summarised in Table 3 statistically significant discriminatory peaks between control PC-3 cells and DMSO extract of propolis-treated PC-3 cells were found to be the proteomic features at m/z 5143, 8703, 12661, 20184 and 32794, detected by CM10 ProteinChip, and the peak at m/z 3772, detected by Q10 ProteinChip. The expression level of the discriminatory proteomic feature with m/z 8703 is shown in Figure 1. The visual comparison of intensities of the peak at 8703 between DEP-treated and untreated PC-3cell lines are shown as box plot display in Figure 2.

**Table 3:** Expression Difference Mapping between DEP-treated and untreated PC-3 cancer cell lines.

Protein Chip	Proteomic Feature [mean m/z (± SD)]	p value	Control PC-3 cells			DEP-treated PC-3 cells		
			Intensity Average	Intensity SD	Intensity CV%	Intensity Average	Intensity SD	Intensity CV%
**CM10**	5143 (1.9)	.0433	9.6	2.7	27.9	7.1	0.6	8.8
	8703 (1.0)	.0433	22.0	3.2	14.3	32.5	8.2	25.4
	12661 (5.4)	.0209	13.2	3.4	25.8	14.6	2.8	19.0
	20184 (4.8)	.0433	1.6	0.8	53.7	0.7	0.2	29.1
	32794 (23.7)	.0209	0.3	0.0	7.1	0.2	0.0	18.1
**Q10**	3772 (2.1)	.0209	4.5	1.3	29.1	5.9	0.5	7.8

DEP refers to DMSO (100%) extract of propolis; WEP refers to water extract of propolis.

### Expression Difference Mapping between water extract of propolis-treated and untreated PC-3 cancer cell lines by SELDI-TOF-MS

As shown in Table 4, statistically significant discriminatory peaks between control PC-3 cells and water extract of propolis-treated PC-3 cells were found to be the proteomic features at m/z 15846,16052 and 24658, detected by CM10 ProteinChip and the peaks at m/z 10348, 10899 and 11603, detected by Q10 ProteinChip.

**Table 4:** Expression Difference Mapping between WEP-treated and untreated PC-3 cancer cell lines.

Protein Chip	Proteomic Feature [mean m/z (± SD)]	p value	Control PC-3 cells			WEP-treated PC-3 cells		
			Intensity Average	Intensity SD	Intensity CV%	Intensity Average	Intensity SD	Intensity CV%
**CM10**	15846 (1.4)	.0433	8.1	1.6	19.2	10.1	1.2	11.6
	16052 (3.5)	.0209	3.3	0.5	13.9	4.2	0.6	14.6
	24658 (3.9)	.0433	1.3	0.2	17.2	1.9	0.5	28.2
**Q10**	10348 (4.4)	.0433	76.5	1.6	2.0	82.0	3.8	4.6
	10899 (6.8)	.0433	8.5	1.7	20.1	9.8	2.6	26.4
	11603 (1.4)	.0209	17.3	0.7	4.0	14.8	1.8	27.1

DEP refers to DMSO (100%) extract of propolis; WEP refers to water extract of propolis.

**Figure 3:**
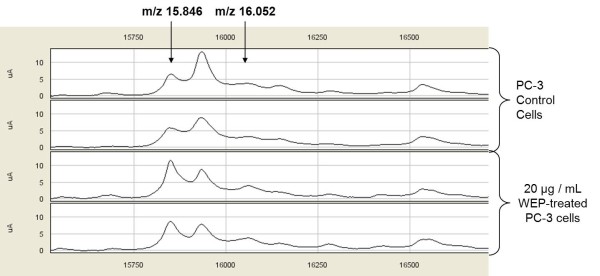
**SELDI-TOF mass spectra of WEP-treated and untreated PC-3 cells by CM10 ProteinChip**. Protein peaks of the peaks 15846 m/z, and 16052 m/z that are positively correlated with WEP treatment of PC-3 cells.

**Figure 4:**
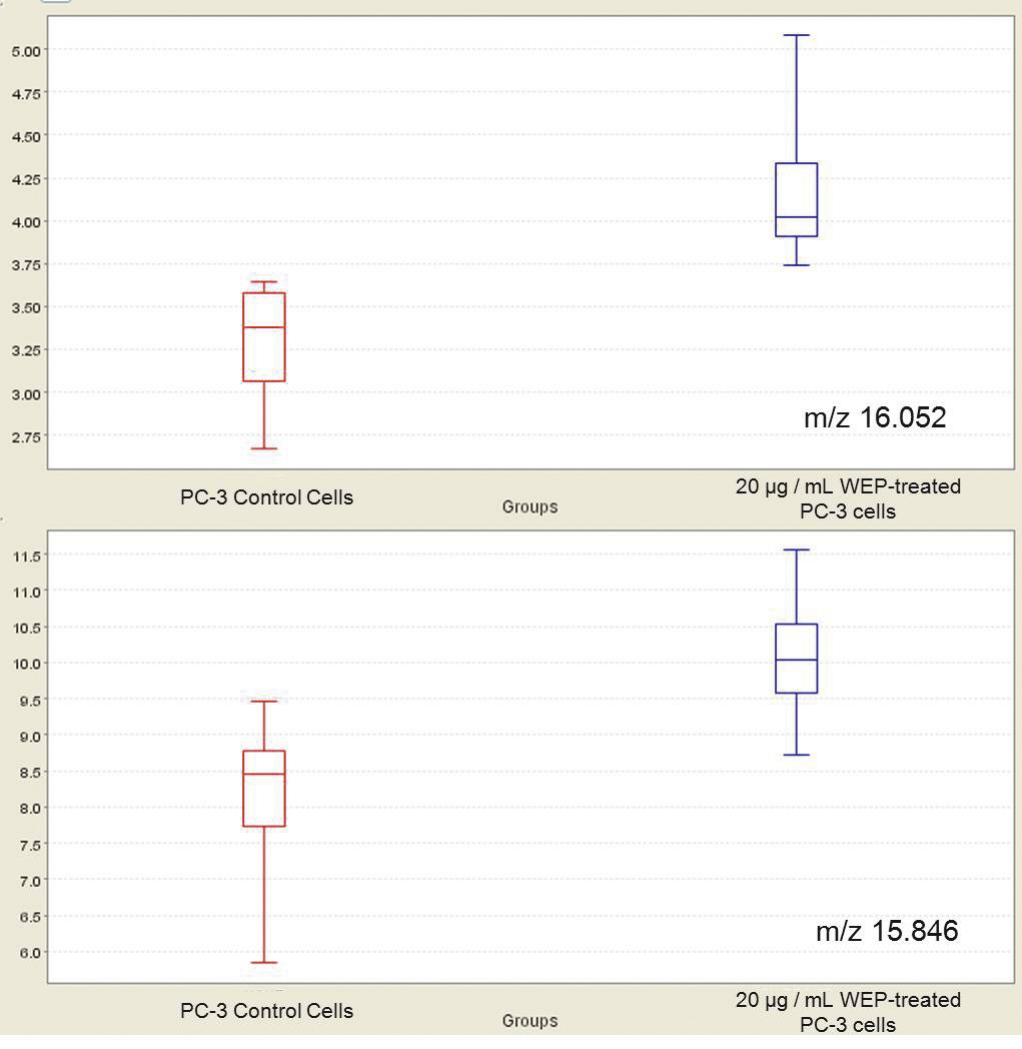
**Box plot displays of intensity levels of 15846 m/z, and 16052 m/z between WEP-treated and untreated PC-3 cells by CM10 ProteinChip**. The comparison of WEP-treated PC-3 cell lysates and control PC-3 cell lysates are performed by using non-parametric Mann Whitney method with Ciphergen Express software, version 3.0.

**Figure 5:**
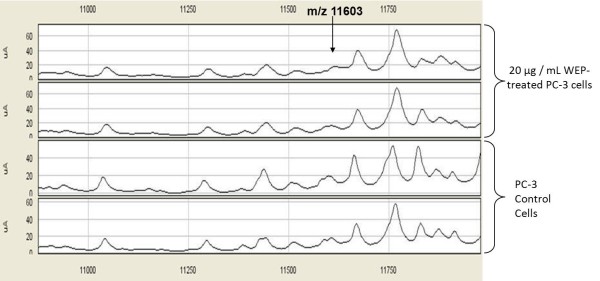
**SELDI-TOF mass spectra of WEP-treated and untreated PC-3 cells by Q10 ProteinChip**. Protein peaks of the peak 11603 m/z, and that positively correlated with WEP-treated PC-3 cells.

**Figure 6:**
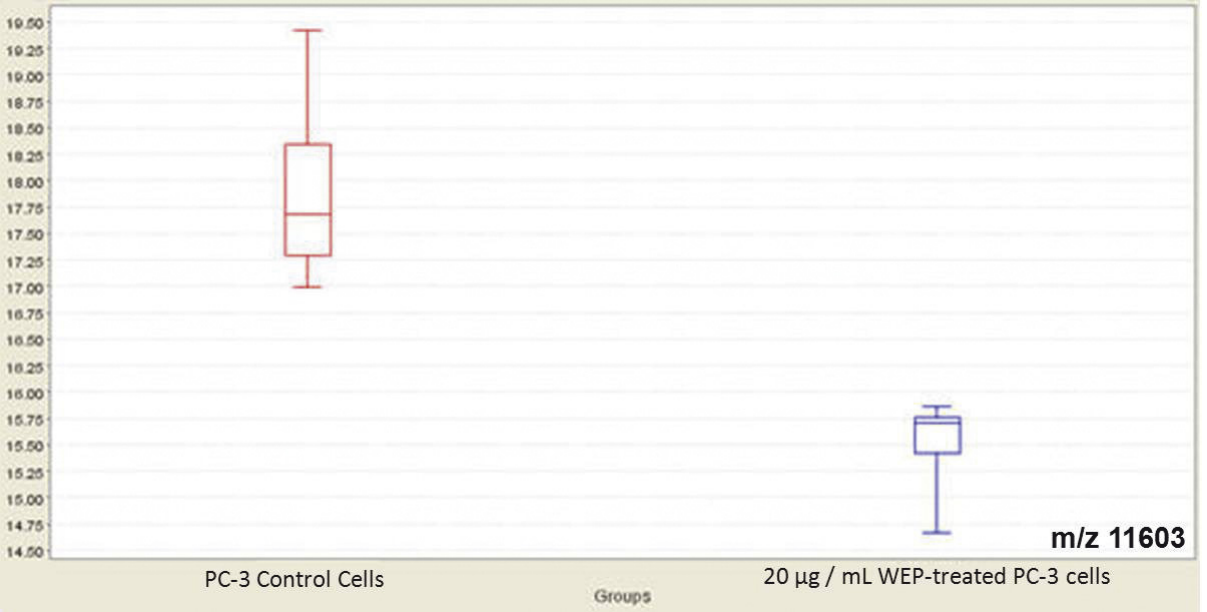
**Box plot displays of intensity levels of 11603 m/z between WEP-treated and untreated PC-3 cells by Q10 ProteinChip**. The comparison of WEP-treated PC-3 cell lysates and control PC-3 cell lysates are performed by using non-parametric Mann Whitney method with Ciphergen Express software, version 3.0.

Intensities of these selected peaks in profiling data were changed by treatment with propolis extracts. The expression levels of two of these significantly discriminatory proteomic features with m/z 15846 and 16052 are shown in Figure 3. The visual comparison of intensities of these two peaks at 15846 and 16052, between WEP-treated and untreated PC-3 cell lines are shown as box plot display in Figure 4. The expression level of the significantly discriminatory proteomic feature with m/z 11603 is shown in Figure 5. The visual comparison of intensities of the peak at 11603 between WEP-treated and untreated PC-3 cell lines are shown as box plot display in Figure 6.

## Discussion

Diet and dietary factors play an important role in preventing and treating chronic diseases including cancer [5,25]. Depending on their isolation step, plant products have been defined as food, food supplement, functional food and nutraceuticals. Pure extracted phytomolecule is named as nutraceuticals, whereas semipurified plant product is named as functional food.

Micronutrients, polyunsaturated fatty acids, and secondary metabolites such as glucosinolates, flavonoids, polyphenols, phytoestrogens, phytosterols, lignans, terpenes and phytates are components of these plant foods [26].

Curcumin (turmeric), capsaicin (green chilies), epigallocatechingallate (green tea), gingerol (ginger), genistein (soya beans), resveratrol (grapes), caffeic acid phenyl ester (propolis from honey bee), sulforaphane (cruciferous vegetables), silibinin, indole-3-carbinol (cabbage), apigenin (tea, cabbage, garlic), allicin (garlic), lycopene (tomatoes), quercetin (rhododendron cinnabarium), and β-carotene are some of the phytochemicals that are related to tumor prevention [26].

Thus, natural and synthetic compounds that can be used in the prevention and/or treatment of cancer are the targets of researchers [25]. The beehive products such as honey, propolis, pollen and royal jelly may be included into functional foods [5].

Reactive oxygen species (ROS) together with other factors are responsible for cellular aging, cell signalling, stress responses, cell proliferation, and many conditions such as cardiovascular diseases, diabetes, arthritis, Parkinson disease, Alzheimer and cancer development [27,28].

The antioxidants serve as a defensive factor against free radicals; thereby protect lipids and other compounds during oxidative damage. Propolis has been shown to be capable of scavenging free radicals through their pharmacologically active constituents such as flavonoids [27]. Thus, propolis is thought to improve health and prevent diseases such as inflammation, heart disease, diabetes and cancer by its antioxidant potential [2]. Anti-tumoral activity of propolis might be attributed to a single substance, or to synergistic effects of several compounds, or to potential metabolites [10].

Aliyazıcıoğlu et al. found that fluorescence positivity decreased (between 3.8% and 11.8%) as concentrations of both dimethyl sulfoxide (DMSO) extracts of propolis and pollen increased for K-562 cell culture, but unchanged (between 20% and 83%) for mononuclear cell (MNC) culture by intracellular dichlorofluorescence (DCFH) test by using flow-cytometric fluorescence analysis. They concluded that DMSO extracts of pollen and propolis inhibited the respiratory burst within cancer cell lines probably by their antioxidant potentials [28].

It has been reported that various activities of propolis may be attributed to a synergism between phenolic (flavonoids, aromatic acids and esters) and other compounds in the resin [7,9]. Thus we preferred to use whole DMSO and water extracts of propolis, rather than its constituents for our study.

Dimethyl sulfoxide (DMSO) is an amphipathic molecule and soluble in both aqueous and organic media. Due to its physiochemical properties, DMSO is a very efficient solvent for water-insoluble compounds and has been used successfully in the treatment of dermatological, urinary, pulmonary, rheumatic and renal manifestations of amyloidosis. Basically through its anti-inflammatory and reactive oxygen species scavenger actions, its use has been purposed in several gastrointestinal diseases [29].

Banskota et al. showed that methyl alcohol (MeOH) extract of the Netherlands propolis had anti-proliferative activity toward highly liver-metastatic murine colon 26-L5 carcinoma (EC_50_, 3.5 mg/mL). They also showed that the compounds isolated from the MeOH extract (benzyl, phenethyl and cinnamylcaffeates) possessed potent anti-proliferative activities with EC_50 _values of 0.288, 1.76 and 0.114 mM, toward colon 26-L5 carcinoma. Thus, they concluded that anti-oxidative activity of these caffeates may play an important role in their anti-proliferative activities [12].

Russo et al. found that compounds obtained in ethanolic extract of Chilean propolis such as galangin, caffeicacid, p-cumaric acid, ferulic acid and caffeic acid phenethyl ester (CAPE) by HPLC analysis exhibited DPPH (2,2-diphenyl-1-picrylhydrazyl) free radical scavenging and superoxide scavenging activity in a dose-dependent manner. It was showed that Chilean propolis exhibited anti-proliferative activity toward KB cells, Caco-2 and DU-145 cells. The cell viability was found in the propolis extract treated-KB, -Caco-2 and -DU145 cells as 9%, 45% and 23%, respectively. They suggested that the anti-proliferative activity of ethanolic extract of Chilean propolis might be mediated by its ability to modulate intracellular reactive oxygen species levels [30].

In the study of Li et al. the effects of ethanolic extracts of Brazilian propolis group l2 and bud resins of botanical origin (*B. dracunculifolia*), and propolis group 3 on proliferation of metastasis (DU145 and PC-3) and primary malignant tumor (RC58T/h/SA#4)-derived from human prostate cancer cells were evaluated. They found that the strongest inhibition was observed in propolis group 3 (sample #3) extracts whereas moderate growth inhibition was observed in human prostate epithelial cells. They also found that propolis group 3 (sample #3) induced growth inhibition (the cells died at 20 μg/mL treated cells) that was associated with G2 arrest and showed induction of p21 expression but no inhibition of cyclin D1, CDK4 (Cyclin-dependent kinase 4) and cyclin B1 expression. In the RC58T/h/SA#4 cells, they found that resins of botanical origin of propolis group 12 (sample #1) and propolis group 12 (sample #2) induced growth inhibition that was associated with S phase arrest and showed inhibition of cyclin D1, CDK4 and cyclin B1 expression [31].

Carballo et al. found that Cuban propolis shows cytotoxicity in the range of 5-23 μg/mL without cross resistance in both wild-type and chemo-resistant human tumor cell lines comprising colon, ovarian, and prostate carcinomas (10 μg/mL and 12.3 μg/mL in PC-3 and LNCap) as well as neuroblastoma. They assumed that plukenetione A which was identified for the first time in Cuban propolis, contributes to the anti-tumoral effect of Cuban propolis mainly by targeting topoisomerase I as well as DNA polymerase. They also observed that some components of propolis were more or less active in the isolated form compared with the whole substance and evidenced that there was a multiple interaction (e.g., synergism, antagonism) between propolis constituents [10].

Wang et al. demonstrated that inhibition of cell growth and induction of apoptosis in PC-3 cells was significantly greater in the combination group (isoflavone and curcumin) than that could be achieved by either agent alone. They also found that the effects of those compounds were associated with decreased Notch-1 expression and DNA binding activity of nuclear factor kappa B (NF-κB) and its target genes such as Cyclin D1, Bcl-2, and Bcl-xL [32].

In our study, DMSO (100%) extract of propolis was found to be more rich in polyphenols and flavonoids according to water extract of propolis and the antioxidant potentials of extracts were found in correlation with the amount of total phenolic compounds in them.

In this study, we wanted to assess the effect of DMSO and water extracts of propolis on viability of PC-3 cancer cell line by using MTT test. DMSO extracts of propolis at final concentrations of 5, 10, 20 μg/mL reduced the cell viability more than those of DMSO solutions. It was shown that water extract of propolis at concentration of 20 μg/mL had the most cytotoxic activity against PC-3 cell lines. Our results suggest that anti-proliferative effect of propolis extracts might be mediated by their anti-oxidant potentials. According to cell viability results, 20 μg/mL concentrations of propolis extracts were chosen to study expression difference mapping by SELDI-TOF-MS.

Nair et al. observed that quercetin, flavonoid found in many fruits and vegetables, and also in propolis, significantly inhibited the growth of the highly aggressive PC-3 prostate cancer cell line and the moderately aggressive DU-145 prostate cancer cell line, whereas it did not affect colony formation by the poorly aggressive LNCaP prostate cancer cell line or the normal fibroblast cell line BG-9. They found that quercetin significantly down-regulated the expression of specific oncogenes and genes controlling G1, S, G2, and M phases of the cell cycle and up-regulated the expression of several tumor suppressor genes [33].

In the study of Cheng et al. anti-tumor mechanism of *RhizomaParidis *total saponin *(*RPTS, a component of herb *RhizomaParidis*) in HepG2 cells was examined by a proteomic analysis. They found a significant change between control (0.01% DMSO) and RPTS (IC_50 _approximately 10 μg/mL) treated cells after 48 h. Among twelve proteins that had been identified by MALDI-TOF-MS (Matrix Assisted Laser Desorption Ionisation - Time of Flight), six proteins were down-regulated (dUTPase, hnRNPK, GMPsynthase, etc.) and six proteins were up-regulated (DNasegamma, Nucleosidediphosphate kinase A, Centrin-2, etc.) by RPTS treatment in HepG2 cells [34].

Lee et al. suggested that caffeic acid phenethyl ester (CAPE), a chemopreventive phytochemical derived from honeybee propolis, suppressed SK-Hep1 cell invasion in a dose-dependent manner by abolishment of matrix metalloproteinases (MM2 and MM9) which are associated with the invasive phenotypes of cancer cells and inhibition of NF-κB DNA-binding activity [35].

Bottoni et al. investigated ciglitazone [PPARγ (peroxisome proliferators-activated receptor γ) agonist]-induced differentiation of a human hepatocarcinoma HepG2 cell line, by monitoring cellular parameters of cytodifferentiation and modifications of cellular protein profiles through 2-DE (Two dimensional electrophoresis) and MALDI-TOF analysis. They found that ciglitazone is a strong differentiating agent for the HepG2 cell line and the proteins of which expression profiles changed, related to cell antioxidant systems, the cell cycle apparatus, signal transduction pathways, cellular stress and invasiveness [36].

Lee et al. showed that sulforaphane (SFN) which is an isothiocyanate found in cruciferous vegetables, exerted cytotoxicity and increased TUNEL (Terminal dUTP nick end labeling) positive cells in a concentration-dependent manner in LNCaP prostate cancer cells. In their study, levels of nine proteins including tubulin β-2, phosphoglucomutase-3 (PGM3), melanoma-derived leucine zipper containing extra-nuclear factor, activin A type I receptor precursor, smoothelin-A, KIA0073, hypothetical protein LOC57691 and two unnamed proteins changed over 8 folds in SFN treated LNCaP cells compared to untreated control by using MALDI-TOF [21].

Szlıszka et al. demonstrated that TRAIL (tumour necrosis factor-related apoptosis-inducing ligand)-resistant prostate cancer cells were sensitized by treatment of ethanolic extract of Brazilian green propolis (EEP) by enhancing the expression of TRAIL-R2 and the activity of NF-κB in LNCaP cells [20].

In this study, statistically significant discriminatory peaks between control PC-3 cells and DMSO extract of propolis-treated PC-3 cells were found to be the proteomic features at m/z 5143, 8703, 12661, 20184 and 32794, detected by CM10 ProteinChip, and the peak at m/z 3772, detected by Q10 ProteinChip (Table 3). Between control PC-3 cells and water extract of propolis-treated PC-3 cells, statistically significant discriminatory peaks were found to be the proteomic features at m/z 15846, 16052 and 24658, detected by CM10 ProteinChip and the peaks at m/z 10348, 10899 and 11603, detected by Q10 ProteinChip (Table 4). Further study is required to identify what those proteins are.

## Conclusions

It was concluded that DMSO and water extracts of Turkish propolis might have anti-proliferative activity through differentiating protein expression profile in PC-3 prostate cancer cell lines along with their antioxidant capacity.

## Methods

### Materials

Dimethylsulfoxide, Sodiumcarbonate, Folin reagent, gallicacid, ethanol, aluminium nitrate, potassiumacetate, quercetin, NaH_2_PO_4_.2H_2_O, Na_2_HPO_4_.2H_2_O, potassiumferricyanide, trichloroaceticacid, iron(III) chloride, trolox, TAC kit, DMEM/Ham's F12 + L-glutamine, penicillin + streptomycin(GIBCO), FBS (FetalBovine Serum) (SIGMA), Bovine Serum Albumin (SIGMA), ethylenediaminetetraaceticacid (EDTA), Trypan Blue, 3-(4,5-Dimethylthiazol-2-yl)-2,5-diphenyltetrazoliumbromide, 4-(2-hydroxyethyl)-1-piperazine ethanesulfonicacid, glycerol, Triton X-100, urea, Protease Inhibitor Coctail (SIGMA), 3-((3-Cholamidopropyl)dimethylammonio)-1-propanesulfonate, Dithiothreitol, Tris, Ammonium acetate, trifluoroaceticacid, acetonitrile, RPMI 1640 (1× + L-Glutamine), ProteinChipAll-in-One-Protein Standart II (Bio-Rad), NP20, H50, IMAC30, CM10 (#C57-30075) and Q10(#C57-30080) ProteinChipArrays (Bio-Rad), SPA (sinapinicacid) (Bio-Rad, SPA0705161) were used in this study.

### Propolis Origin

Propolis samples which were produced by honey-bees (Apismellifera L.) in various regions of Turkey were provided by Trabzon Agricultural Development Cooperative and mixed.

### Preparation of Dimethylsulfoxide (DMSO) and Water Extract of Propolis

Propolis sample was frozen in -80°C and grated. The grated propolis sample was frozen in-80°C again and grinded (Retsch, ZM 200). 5 g portions of grinded propolis were dissolved in 20 mL of DMSO (100% w/v) and water by continuous mixing at 150 rpm and 60°C for 24 h in shaking incubator (Shelleb/Sheldon Mod:514, USA). After incubation extracts were filtered and centrifuged (Eppendorf centrifuge 5810 SN: 11259) at 4000 rpm for 10 min. Collected supernatants were mixed and stored at +4°C in dark. Then working extracts of propolis at concentrations of 1.25, 2.5, 5.0 and 12.5 mg/mL were prepared by diluting with water to measure total polyphenol content, total flavonoid content, ferric reducing antioxidant potential (FRAP) and total antioxidant capacity (TAC).

### Phenolic Content and Antioxidant Potentials of Propolis Extracts

#### Determination of Total Polyphenol Content

Total polyphenol content of propolis extracts was determined spectrophotometrically according to the modified Folin-Ciocalteu method [37]. The method was adapted to the 96 well microplatereader. Total phenols in the propolis extracts were expressed as gallic acid equivalents, using a standard curve of freshly prepared gallic acid solutions.

#### Determination of Total Flavonoid Content

Total flavonoid content of propolis extracts was determined spectrophotometrically by modified aluminum nitrate colorimetric method that adapted to the 96 well microplatereader [27]. Total flavonoid content of propolis extracts were expressed as quercetin equivalents, using a standard curve of freshly prepared quercetin solutions.

#### Determination of Ferric Reducing Antioxidant Potential (FRAP)

The reducing power of propolis extracts was determined by the method that is based on ferric to ferrous ion reduction at low pH [27]. The method was adapted to the 96 well microplatereader. Antioxidant potentials of propolis extracts were expressed as trolox equivalents, using a standard curve of freshly prepared trolox solutions.

#### Determination of Total Antioxidant Capacity (TAC)

Total antioxidant capacity of propolis extracts was determined according to a novel colorimetric method optimised by Erel Ö [38]. TAC was determined by using TAC kit and the assay results are expressed in mmolTrolox/100 gpropolis.

#### MTT-cell viability assay

PC-3 (ATCC, CRL-1435) cancer cell line was obtained from the Department of Haematology, Faculty of Medicine, GATA, Ankara. PC-3 cells were maintained in RPMI 1640 and supplemented with 10% FBS, 1% penicillin and streptomycin in T-75 cm^2 ^flasks at 37°C and 5% CO_2 _atmosphere. The cell viability was estimated by MTT [3-(4,5-dimethylthiazol-2-yl)-2,5-diphenyltetrazolium bromide] assay, which depends on the reduction of MTT by the mitochondria of living cells to form a blue formazan product [39]. Then, cells were plated in DMEM/F:12 medium at a density of 1 × 10^4 ^cells/well in 96 flat-bottomed well plates. After 24 h plating, test extracts were added at final concentrations of 5, 10 and 20 μg/mL DMSO and water extracts of propolis and 0.008%, 0.004%, 0.002% DMSO/mL (Extracts were diluted with RPMI 1640). After 24 h incubation, the medium was replaced with MTT, for further 4 h incubation. Then the MTT-formazan was solubilised in DMSO/Ethanol (1:1) and the optical density was measured at a wavelength of 570 nm in microplatereader. The experiments were repeated 8 times.

#### Incubation of PC-3 cancer cell lines with DMSO and water extracts of propolis

According to MTT-cell viability assay results, PC-3 cancer cell lines were incubated with DMSO and water extracts of propolis at final concentration of 20 μg/mL. PC-3 cancer cell lines which did not contain extract were used as control cells. PC-3 cells were incubated for 24 h in DMEM/F:12 supplemented with 1% BSA, 1% penicillin and streptomycin in T-75 cm^2 ^flasks at 37°C and 5% CO_2_ atmosphere. The experiments were done thrice.

#### Lysis of PC-3 cancer cell lines after incubation

After 24 h incubation with water and DMSO extracts of propolis, treated and untreated PC-3 cells were lysed by modified HNTG cell lysis procedure [40]. The DMEM/F:12 medium was removed and cells adhered to flask were rinsed twice with 5 mL of Phosphate Buffered Saline (PBS, pH 7.4) to remove BSA. Then, cells were rinsed with 5 mL of ice cold HNG (25 mM Hepes, 25 mM NaCl, 10% glycerol, pH 7.5). HNG was completely removed by aspiration and 500 μL of HNTG lysis buffer [(25 mM Hepes, 25 mM NaCl, 10% glycerol, 0.1% Triton X-100, Protease Inhibitor Coctail (1:1000), pH 7.5] was added on cells to lysis them and then they were incubated for 10 min on ice. Lysed cells were transferred to 1.5 mL eppendorf and incubated in thermomixer at 150 rpm and 4°C and centrifuged for 10 min at 14.000 g in microcentrifuge. The supernatants which contain cell proteins were collected separately and aliquoted and stored at -80°C until used.

#### Expression Difference Mapping (Proteomic profiling) using SELDI-TOF mass spectrometry

To determine the best protein profiles regarding number and resolution of the protein peaks, four chips with different ProteinChip surfaces [cationic (CM10), anionic (Q10), hydrophobic (H50), and Cu metal binding (IMAC30), CiphergenBiosystems, Fremont, CA, USA] were tested. Weak cation exchange (CM10) and strong anion exchange (Q10) protein chip were selected for further analysis, which displayed the best protein profile. Mass accuracy was calibrated externally by using All-In-One peptide and All-In-One protein molecular mass standard (CiphergenBiosystems). All-in-One-Protein Standard II consisted of [Hirudin, recombinant (6,964 Da)], [Cytochrome C (bovine) (12,230 Da)], [Myoglobin (equine) (16,951 Da)], [Carbonic anhydrase (bovine red blood cells (RBC) (29,023 Da)], [Enolase (*S. cerevisiae*) (46,671 Da)], [Albumin (bovine) (66,433 Da)] and [IgG (bovine) (147,300 Da)]. The mass spectra of proteins were generated by using an average of 175 laser shots at a laser intensity of 225 to 250 arbitrary units and laser energy of 7000 nJ. To stabilize variation in sample loading, the data was further normalized to total ion current and aligned. Analyses included automatic peak detection, baseline subtraction and mass accuracy calibration.

Each of three samples of untreated PC-3 cell lysates, DMSO extract of propolis (DEP)-treated PC-3 cells and water extract of propolis (WEP)-treated PC-3 cells were loaded to CM10 and Q10 ProteinChips. Samples were analyzed in duplicate with the SELDI Protein-Chip system (PCS 4000 Systems-CiphergenBiosystems) to obtain a proteomic profile with molecular masses ranging from 0.3 to 50 kDa. Resulting chip data was analysed by using Ciphergen Express Software v.3.0.

#### Weak cation exchange ProteinChip (CM10 chip)

The array spots were first pre-equilibrated twice with 150 μL of binding buffer (50 mM ammonium acetate (pH 4) + 0.1% Triton X-100) for 5 min at room temperature. Then, 90 μL of cell lysate which was denaturated with DTT containing denaturation buffer (9 M urea, 2% CHAPS, 2 mM DTT, 150 mM Tris-HCl, pH 9) and diluted (1:3) in the binding buffer, was applied to CM10 ProteinChip spot in duplicate and incubated for 1 h at room temperature. After incubation, each array was washed three times with 150 μL of binding buffer (5 min), and rinsed once with 200 μL of deionized water. After air drying, 1 μL of saturated solution of sinapinic acid (SPA) in 50% acetonitrile: 0.5% TFA (v/v) was applied on spots and allowed to air dry. The mass spectra of proteins were analysed by the principle of time-of-flight (TOF) using SELDI-TOF MS.

#### Strong Anion exchange ProteinChip (Q10 chip)

For the detection of protein profiles of the samples using Q10 ProteinChip Array, 150 μL of binding buffer (50 mM Tris-HCl, pH 8) was added to each spot and incubated for 5 min at room temperature. The above steps were repeated twice. Then, 90 μL of cell lysate diluted (1:3) in the binding buffer before, was applied to Q10 ProteinChip spot in duplicate and incubated for 1 h at room temperature. After incubation, it was washed three times with 150 μL of the binding buffer and then rinsed once with 200 μL of deionized water. Finally, after air drying, 1 μL of saturated solution of SPA was added to each protein-loaded spot and allowed to air dry. The mass spectra of proteins were analysed using SELDI-TOF MS.

### Statistical analysis

Protein peaks were clustered with the Ciphergen Express software, version 3.0 performing Expression Difference Mapping (EDM). Proteomic features were labeled with 5.0 S/N (signal to noise ratio), 3 valley depth for the first pass; minimal peak threshold: 20% of all spectra and 3.0 S/N and 1 valley depth for the second pass with 0.3% cluster mass window. Discriminatory peaks were identified using the Mann-Whitney non-parametric test depending on peak intensities. Statistically significant discriminatory peaks between groups were determined with area under receiver operating characteristics curves (AUROC) > 0.8 and p < 0.05.

## Abbreviations

CAPE: Caffeic acid phenethyl ester; CDK4: Cyclin-dependent kinase 4; CM10 ProteinChip: Weak cation exchange ProteinChip; DCFH: Dichlorofluorescein; DEP: Dimethyl sulfoxide extract of propolis; DPPH: 2,2-diphenyl-1-picrylhydrazyl; EDM: Expression difference mapping; FRAP: Ferric reducing antioxidant potential; MALDI-TOF: Matrix assisted laser desorption ionisation-time of flight; MM: Matrix metalloproteinase; MNC: Mononuclear cell; NF-κB: Nuclear factor kappa B; Q10 ProteinChip: Strong Anion exchange ProteinChip; PMFs: Peptide Mass Fingerprint; PPARγ: Peroxisome proliferators-activated receptor γ; ROS: Reactive oxygen species; RPTS *:RhizomaParidis *total saponin; SELDI-TOF-MS: Surface-enhanced laser desorption ionization time-of-flight mass spectrometry; TAC: Total antioxidant capacity; TRAIL: Tumour necrosis factor-related apoptosis-inducing ligand; TUNEL: Terminal dUTP nick end labeling; WEP: Water extract of propolis; 2-DE: Two dimensional electrophoresis.

## Competing interests

The authors declare that they have no competing interests.

## Authors' contributions

YB and MÇ carried out the preparation of propolis extracts, determination of phenolic content and antioxidant potentials of extracts and MTT. YB carried out the incubation of cells with extracts and lysis of incubated cells. YB and SCK carried out the expression difference mapping and performed statistical analysis. YB, OD and MB conceived of the study, and participated in its design and coordination. YB, SCK and OD drafted the manuscript. All authors read and approved the final manuscript.
